# A practical guide to the diagnosis and management of osteoporosis in childhood and adolescence

**DOI:** 10.3389/fendo.2023.1266986

**Published:** 2024-01-25

**Authors:** Leanne M. Ward

**Affiliations:** ^1^ Department of Pediatrics, Faculty of Medicine, University of Ottawa, Ottawa, ON, Canada; ^2^ Department of Pediatrics, Children's Hospital of Eastern Ontario, Ottawa, ON, Canada

**Keywords:** children, osteoporosis, fractures, osteogenesis imperfecta, chronic illness osteoporosis, bone mineral density, bisphosphonate, pamidronate

## Abstract

Osteoporosis in childhood distinguishes itself from adulthood in four important ways: 1) challenges in distinguishing otherwise healthy children who have experienced fractures due to non-accidental injury or misfortunate during sports and play from those with an underlying bone fragility condition; 2) a preponderance of monogenic “early onset” osteoporotic conditions that unveil themselves during the pediatric years; 3) the unique potential, in those with residual growth and transient bone health threats, to reclaim bone density, structure, and strength without bone-targeted therapy; and 4) the need to benchmark bone health metrics to constantly evolving “normal targets”, given the changes in bone size, shape, and metabolism that take place from birth through late adolescence. On this background, the pediatric osteoporosis field has evolved considerably over the last few decades, giving rise to a deeper understanding of the discrete genes implicated in childhood-onset osteoporosis, the natural history of bone fragility in the chronic illness setting and associated risk factors, effective diagnostic and monitoring pathways in different disease contexts, the importance of timely identification of candidates for osteoporosis treatment, and the benefits of early (during growth) rather than late (post-epiphyseal fusion) treatment. While there has been considerable progress, a number of unmet needs remain, the most urgent of which is to move beyond the monotherapeutic anti-resorptive landscape to the study and application of anabolic agents that are anticipated to not only improve bone mineral density but also increase long bone cross-sectional diameter (periosteal circumference). The purpose of this review is to provide a practical guide to the diagnosis and management of osteoporosis in children presenting to the clinic with fragility fractures, one that serves as a step-by-step “how to” reference for clinicians in their routine clinical journey. The article also provides a sightline to the future, emphasizing the clinical scenarios with the most urgent need for an expanded toolbox of effective osteoporosis agents in childhood.

## Introduction

Fractures in childhood are common, with about half of children experiencing at least one fracture prior to adulthood (1, 2), and more than 20% of children with fractures having sustained a prior broken bone (3). This poses challenges for the pediatric osteoporosis clinician, who must distinguish otherwise healthy children who have experienced non-accidental injury or misfortune during sports and play from those with an underlying bone fragility condition. In some cases, the diagnosis is obvious as the child walks into the assessment room (e.g., strikingly blue sclera), or from the clinical context highlighted in the referral note (e.g., a serious acute or chronic childhood illness). When the diagnosis is not obvious, careful history-taking, thorough medical and musculoskeletal physical examinations, and genetic, laboratory, and imaging evaluations can channel the assessment to an appropriate diagnosis and treatment plan.

In the course of addressing these challenges, it is important to understand that osteoporosis in childhood distinguishes itself from adulthood in a number of important ways, including 1) a preponderance of monogenic “early onset” osteoporotic conditions that unveil themselves during the pediatric years; 2) the unique potential, in those with residual growth and transient bone health threats, to reclaim bone density, structure, and strength without bone-targeted therapy following fragility fractures; and 3) the need to benchmark bone health metrics to constantly evolving “normal targets”, given the dramatic changes in bone size, shape, and metabolism that take place from birth through late adolescence. At the same time, there are a number of diagnosis and management principles that are similar across the lifespan, including the importance of vertebral fractures as a major sign of osteoporosis-related morbidity, and the need for timely identification and treatment of osteoporosis in order to prevent the “fracture, re-fracture cycle” that can lead to a downward health spiral (e.g. femur fractures in boys with Duchenne muscular dystrophy (DMD) and in the elderly leading to permanent loss of ambulation in both settings). Overall, osteoporosis management should be viewed along a continuum, with the stage set in childhood for optimization of bone strength across the lifespan (4).

On this backdrop, the pediatric osteoporosis field has evolved considerably over the last few decades, given the discovery of novel genes implicated in childhood-onset bone fragility and their associated phenotypes, improved understanding of the natural history of bone fragility in the chronic illness setting and associated risk factors, the development of effective diagnostic and monitoring pathways in different disease contexts, evidence for the importance of timely identification of candidates for osteoporosis treatment, and robust clinical trials that have highlighted the impact of bisphosphonate therapy in the pediatric setting. To this day, intravenous (IV) bisphosphonates remain the cornerstone of osteoporosis therapy in childhood, with a first-ever international, randomized, IV placebo-controlled trial of zoledronic acid published in 2021 (5) following decades of use on compassionate grounds in various pediatric contexts.

While there has been considerable progress, a number of unmet needs remain, the most urgent of which is to move beyond the monotherapeutic anti-resorptive landscape to the study and application of anabolic agents that are anticipated to not only improve bone mineral density but also cross-sectional bone diameter in those with gracile bones (such as osteogenesis imperfecta [OI] and neuromuscular disorders). The purposes of this review therefore are two-fold. The first is to provide a *practical guide* to the diagnosis and management of osteoporosis in childhood, one that serves as a step-by-step “how to” reference for clinicians faced with evaluating a child with a history of, or at risk for, fractures. The second is to provide a *sightline to the future*, emphasizing the clinical scenarios with the most urgent need for an expanded toolbox of effective osteoporosis agents in childhood.

## A step-by-step guide to the diagnosis and management of children with a history of fracture(s)

### Step 1: Rule out rickets + non-accidental injury and understand whether the child presents with “undifferentiated bone fragility” or in “a clinical context known to be associated with bone fragility”

**Figure 1:**
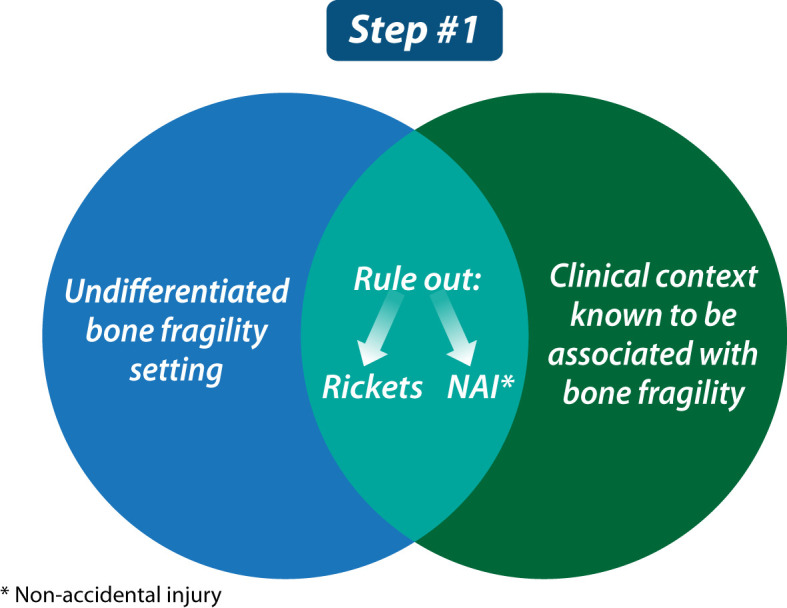
The first step in evaluating a child with bone fragility is to rule out rickets, consider that the child may have been subjected to non-accidental injury, and understand whether the child presents in a clinical context known to be associated with bone fragility or not (the latter, referred to as “undifferentiated bone fragility”).

Step 1 is illustrated in **Figure 1**. Any child presenting with bone fragility may have rickets as the causative etiology, or as a co-morbid condition that exacerbates fracture risk. Therefore, all children referred for an evaluation of fragility fractures should undergo an initial biochemical assessment to rule out rickets including serum calcium, phosphate, creatinine, alkaline phosphatase, parathyroid hormone (PTH), 25-hydroxyvitamin D (25OHD) and urinary creatinine, calcium, and phosphate. 1,25-dihydroxyvitamin D may also be indicated if more rare causes of rickets such as 1-alpha-hydroxylase deficiency or inactivating pathogenic variants of the vitamin D receptor are suspected. This rapid screen should be done soon after referral to the osteology clinic, in order to identify children with bone fragility who, in fact, have an underlying rachitic disorder. Following an assessment to identify urgent, medically-actionable disorders of mineral ion metabolism such as vitamin D deficiency or other forms of rickets, more extensive evaluations that are specifically tailored to the suspected rickets versus osteoporosis scenario can be undertaken.

Unfortunately, non-accidental injury (NAI) is also on the differential diagnosis of any child with a history of fractures; while maltreatment is most common in infants and toddlers, an older child is not exempt. Overall, maltreatment should be suspected if there are delays in seeking medical attention, if the clinical evaluation reveals unexplained bruising or other signs of injury such as retinal or intra-cerebral hemorrhages, if there are multiple fractures in various stages of healing, if physical stigmata of a congenital bone fragility condition are lacking despite significant fractures (e.g., absence of facial dysmorphism, blue sclerae, abnormal teeth, plagiocephaly, enlarged fontanelles, or skeletal deformity), if the mechanism of injury is unknown, or if the reported mechanism of injury does not correlate with the fracture type. Our pediatric osteology clinic recommends a low threshold for genetic testing in children referred under the umbrella of “child protection”, with the goal of ensuring that no child with an underlying monogenic cause of bone fragility is inappropriately labeled as NAI. It is also important to appreciate that a child with NAI may rarely have an underlying bone fragility condition that is unveiled by the maltreatment. Since NAI is at once serious and emotionally charged, and since accurate and timely diagnoses are critical, the child and youth protection team is advised to work closely with multi-disciplinary experts including geneticists, orthopedic surgeons, radiologists, and pediatric osteologists.

It should be noted that rib and scapula “pseudofractures”, otherwise known as “looser zones”, are typical features of rickets and osteomalacia. In cases where rickets and osteomalacia have been effectively ruled out, flat bone fractures (scapula, sternum, skull, and rib) usually arise from significant trauma (i.e., falling off a bike). Flat bone fractures without a known history of major trauma, and in the absence of rickets and osteomalacia, raise red flags for possible non-accidental injury. Exceptions do occur, such as low-trauma skull and rib fractures in infants with OI, emphasizing once again the need for experts in congenital bone fragility to be part of the bone fragility evaluation team.

Once rickets and non-accidental trauma have been ruled out, the next step is to determine whether the child has a congenital bone fragility condition (primary osteoporosis) or an acute or chronic illness known to be associated with an increased risk of fracture (secondary osteoporosis). Since primary and secondary osteoporosis can co-occur, both diagnoses need to be considered even when one or the other appears obvious.

### Step 2: Assess the child for signs of congenital bone fragility (primary osteoporosis) or an acute or chronic illness (secondary osteoporosis)

**Figure 2:**
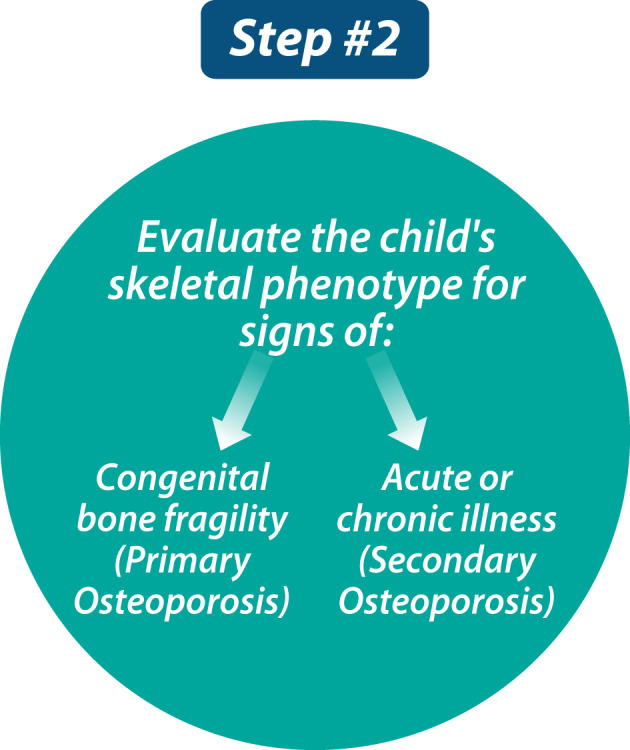
The second step in evaluating a child with bone fragility is to comprehensively assess the child’s skeletal phenotype and, in so doing, search for signs of primary versus secondary osteoporosis.

The approach to Step 2 (**Figure 2**) may be obvious when the child is referred in a specific clinical context (such as leukemia, glucocorticoid-treated diseases, or neuromuscular disorders), with a family history of primary osteoporosis (such as OI), or with already-identified physical stigmata of congenital bone fragility (such as blue sclerae or extreme short stature). Whether clues to the etiology are obvious or not, comprehensive skeletal phenotyping is foundational to this second step, in order to provide information about the potential diagnosis (if not already apparent), to establish the child’s baseline skeletal status (against which treatment effect may be benchmarked), to determine which of the multi-disciplinary services need to be involved in the child’s care (such as orthopedics, neurosurgery, dentistry, rehabilitation medicine, audiology), and to develop goals for the patient’s management (e.g., pain relief, reduced fracture rates, functional mobility).

#### Skeletal phenotyping: history and physical examination

Despite advances in genetic testing and diagnostic imaging (dual-energy x-ray absorptiometry [DXA], peripheral quantitative computed tomography [pQCT], high-resolution pQCT, and increased availability of magnetic resonance imaging), the history and physical examination still provide most of the information required to decide the extent to which a comprehensive skeletal evaluation is necessary and/or to formally diagnose a child with osteoporosis. The history is particularly important to understand the mechanisms of injury resulting in fracture; to this end, some basic “pediatric fracture statistics” are necessary to contextualize the child’s fracture phenotype, as follows.

The risk of fracture between birth and 16 years ranges from 42% to 64% for boys, and from 27% to 40% for girls (2). A consistent finding across all epidemiological studies is that the most frequent site of fracture is the forearm, which accounts for nearly half of all pediatric fractures (2, 6). Sixty-five percent of long bone fractures in childhood involve the upper extremity, and 7% to 28% the lower extremity (2). The fracture rate during childhood is higher than during adult life, hypothesized to result from the constant lag during the growing years between the mechanical challenges that induce bone tissue strain (muscle forces and longitudinal growth) and the necessary adaptive changes in bone structure that foster bone strength in response to tissue strain (7).

Recognizing that long bone fractures are extremely common in childhood, the International Society for Clinical Densitometry (ISCD) 2013 Position Statement defined a significant fracture history as ≥ 2 long bone fractures by age 10 years or ≥ 3 long bone fractures by age 19 years (8). These numbers are reasonable for a child *without* physical stigmata or risk factors for fractures, to avoid over-diagnosis of osteoporosis in a child who has been unlucky during sports or play. However, other factors should also be considered in the decision to initiate a comprehensive bone health evaluation and/or to diagnose a child with osteoporosis: the clinical context in which the child presents with fractures, the degree of trauma, and the location + radiographic features of the fracture(s) are primordial to the assessment.

**Figure 3:**
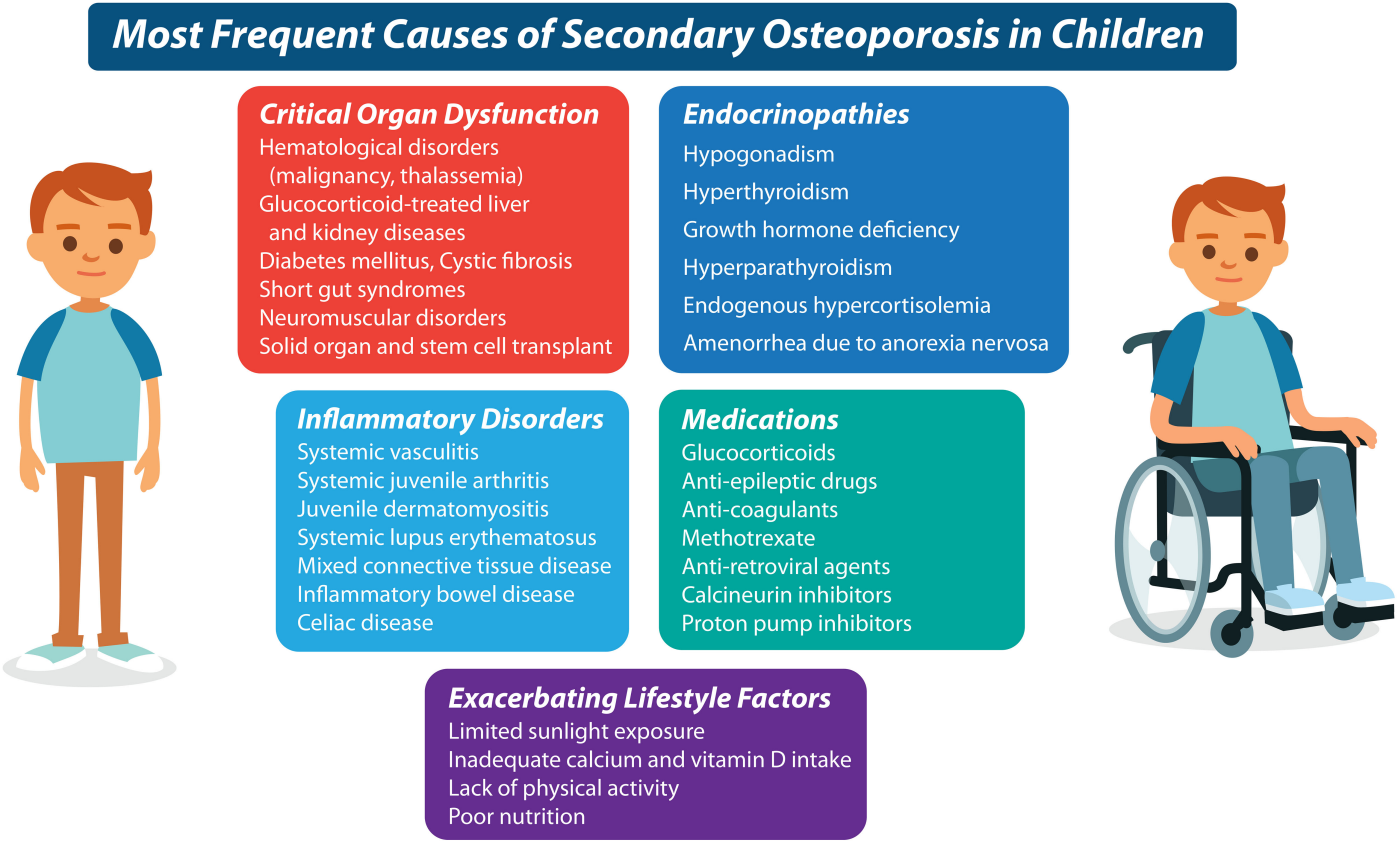
It is now known that numerous underlying conditions can cause or exacerbate a predisposition to bone fragility in the chronic illness setting. The most frequent diseases and their treatments which predispose to secondary osteoporosis in children, are listed here.

The importance of the clinical contexts in which children present with fractures (and associated risk factors) cannot be underestimated. Even a *single long bone* fracture can represent a major osteoporotic event in children with first presentations of OI or other skeletal dysplasias associated with bone fragility (9), and in children with significant risk factors for osteoporosis, such as neuromuscular disorders, leukemia, or other GC-treated conditions (10–12) (**Figure 3**). In settings without a known fracture risk, the degree of trauma associated with the fracture is particularly important in adjudicating the fracture significance. The ISCD (8) has defined low-trauma fractures as those occurring outside of motor vehicle accidents or falling from 10 feet (3 meters) or less. With respect to falls in the high-risk chronic illness setting, a more conservative definition has been used - falling from a standing height or less, at no more than walking speed (13). This latter definition has been shown to hold validity in the pediatric chronic illness setting since vertebral fractures predicted incident low-trauma long bone fractures that were defined in this way (13).

At the same time, it is important to recognize that children with high-trauma fractures may also have a bone fragility condition, a reminder that screening for telltale stigmata, even at the time of first presentation for fracture management in the orthopedic clinic, is important. While femur fractures are particularly concerning, even a single tibia or humerus fracture can represent an osteoporotic event in those at risk and should, therefore, trigger careful evaluation of the injury’s mechanism. Forearm fractures are so common in children that usually recurrent fractures at this site are needed to instigate a more comprehensive bone health evaluation unless the clinical context suggests otherwise. Comminuted fractures and those with atypical displacement are also significant regardless of the long bone site, especially when they occur in the absence of trauma. When in doubt, a multi-disciplinary approach (including an orthopedic surgeon, radiologist, and osteologist) will provide additional insight into the “typical” or “unusual” nature of a given pediatric fracture.

Low-trauma vertebral fractures, both symptomatic and asymptomatic, are a radiographic signature of osteoporosis in both children and adults. To this point, the ISCD endorses that ≥ 1 vertebral fracture, defined as a >20% loss of vertebral height ratio according to the Genant semi-quantitative method (14), is consistent with a diagnosis of osteoporosis in children (15). Pediatric vertebral fractures are extremely rare in the absence of trauma (1), but occur in 75% of children with even mild OI (16), in one-third of children with leukemia (13), in >50% of boys with GC-treated DMD (17), and in 16% of otherwise healthy fracture-prone children (18). In a study of children with leukemia, the positive relationship between Genant-defined vertebral fractures at diagnosis and subsequent new vertebral and long bone fractures provided validity for the use of the Genant method to define vertebral fractures in children (13). The fact that vertebral fractures can be a presenting sign of serious systemic diseases like leukemia and inflammatory disorders underscores the importance of the 2013 ISCD recommendation that even a single low-trauma vertebral fracture can be a manifestation of osteoporosis in children (11, 19, 20). Vertebral fractures should be evaluated by a health care professional with the appropriate trained expertise, including the ability to distinguish vertebral fractures from normal physiological rounding in the thoracic region and from other normal variants (21).

**Figure 4:**
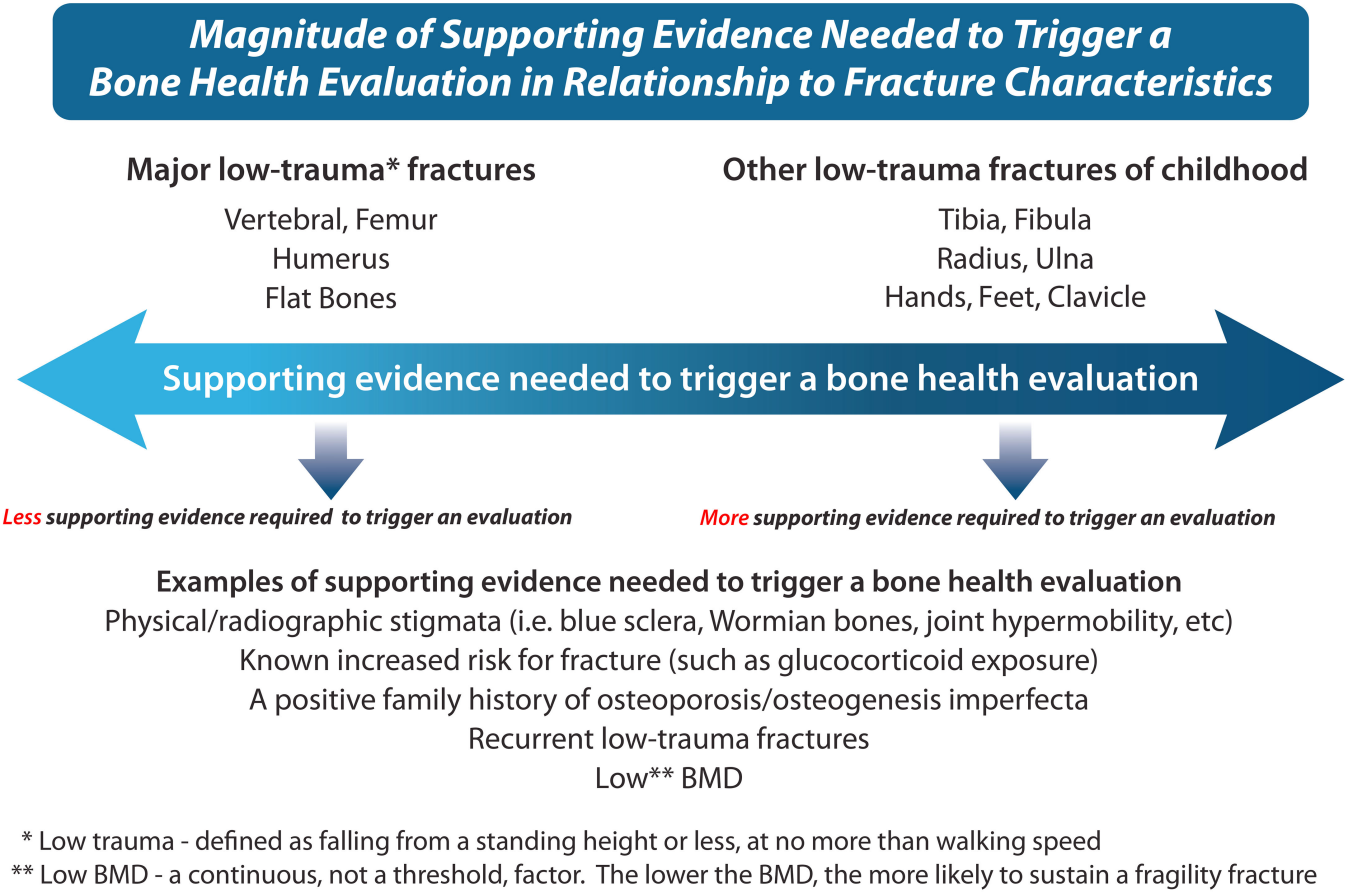
This figure describes the magnitude of supporting evidence that is needed to trigger a bone health evaluation in relationship to fracture characteristics, a key component of the bone fragility assessment. For example, a single low-trauma femur, humerus, or vertebral fracture at presentation means that “ less clinical supporting evidence” is needed to trigger the bone health evaluation, given the seriousness of such fractures. On the other hand, more trivial fractures such as fingers and toes may necessitate “more supporting clinical evidence” to trigger the bone health evaluation (depending on whether there is a clinical context known to be associated with bone fragility, in which case even more trivial fractures may be significant). A comprehensive history, physical examination, and review of the x-ray fracture features provide the necessary information to help guide the decision to proceed with a formal bone health evaluation.

The physical examination in a child presenting with fractures involves assessment of anthropometry and body disproportionality (for skeletal dysplasias associated with bone fragility), as well as other hallmark features such as blue sclera, plagiocephaly, joint hypermobility, skin laxity, scoliosis, limb deformity, tooth abnormalities, easy bruising, facial dysmorphism, multiple Wormian bones, and/or a positive family history. In the presence of one or more of these signs, the threshold for initiating a bone health evaluation is lower than in the absence of these features (**Figure 4**). In children with physical stigmata which together suggest the possibility of a congenital bone fragility disorder, the osteoporosis assessment can be carried out even before presentation with fractures, to pursue a monogenic form of osteoporosis (22), and to detect asymptomatic vertebral fractures (10). In children with history and physical examination features suggestive of an underlying disease, basic laboratory testing with referral to the appropriate specialty (oncology, gastroenterology, rheumatology, neurology, etc.) is warranted. Rarely, both primary and secondary osteoporosis can co-occur in the same patient, as highlighted in a report describing three boys with DMD, two of whom had an additional diagnosis of osteogenesis imperfecta (*COL1A1* pathogenic variants) and a third with a mutation in the *COMP* gene causing pseudoachondroplasia. Therefore, a general rule of thumb is that any child presenting to the osteology clinic with fractures should undergo a thorough review with an open mind to the diagnostic possibilities, even if the diagnosis initially seems obvious.

#### Skeletal phenotyping: diagnostic imaging and genetic testing

Once the decision has been made to proceed with further investigations based on these guiding principles, the next step is to carry out diagnostic imaging and, if appropriate for the clinical context, genetic testing. As for any specialty, the pediatric osteologist should develop a level of comfort reviewing the most frequently-requested imaging including bone age (for skeletal maturation), lateral spine x-rays (or DXA-based “vertebral fracture assessment”) for vertebral fractures (15), and a skeletal survey (in suspected skeletal dysplasias for basic radiographic signs including Wormian bones, scoliosis, kyphosis, lordosis and limb deformity; additional features may require the assistance of a radiologist, depending on the clinician’s level of training and expertise). BMD is also an important component of the evaluation, albeit adjuvant due to the lack of sensitivity and specificity for a given osteoporotic condition. Additional challenges in the use of DXA-based BMD as a diagnostic tool have been discussed extensively elsewhere (23), and are recapitulated in brief here. A comprehensive description of bone imaging assessment techniques in pediatric osteoporotic conditions can be found in a recent review (24).

While a low DXA-based BMD raises the index of suspicion for an osteoporotic fracture, it is not diagnostic, since BMD can be low simply due to a size artifact (as in short stature), or in non-osteoporotic conditions with fractures such as rickets. Furthermore, BMD by DXA can be “normal” (i.e. Z-score -2 to +2) in children with fractures due to both primary and secondary osteoporosis. Broadly speaking, BMD is only one of many jigsaw pieces that orient the clinician as to whether there are sufficient clinical features to warrant expanded diagnostic testing, such as genetic profiling for primary osteoporosis, or chronic illness assessments. The main purpose of BMD in the childhood fracture setting, then, is to provide additional supporting evidence to justify a more comprehensive osteoporosis work-up in equivocal cases. In uncertain cases, the BMD trajectory can be useful, with a loss of ≥ 0.5 SD considered to be clinically significant, providing a threshold to trigger more comprehensive bone health testing (25). The clinical utility of DXA results when based on cross-sectional assessment alone is challenging, since BMD Z-scores generated by different normative data, even after undergoing DXA machine cross-calibration, can vary by as much as 2 standard deviations (an observation which invalidates the use of a Z-score threshold to trigger care pathways) (26).

A number of other considerations are necessarily taken into account when acquiring and interpreting DXA scans in children. The choice of skeletal site should be informed by individual patient characteristics, and local access to appropriate reference data is paramount. Lumbar spine (L1-L4) and whole body (total body less head) BMD have been the most widely used parameters in children to date and are associated with fracture risk (13, 27). In 2019, the ISCD recommendations were updated to additionally endorse DXA-based BMD at the distal forearm, proximal hip, and lateral distal femur in children who need additional information for clinical decision-making, or in whom spine or whole body DXA scans cannot be obtained (e.g. indwelling hardware) (15). Areal BMD by DXA is subject to size artifact; therefore, children with short stature and/or pubertal delay will have artificially low BMD Z-scores relative to healthy reference data. To better estimate BMD in short children, size-adjustment techniques have been developed including bone mineral apparent density (28, 29), and height Z-score-adjusted BMD Z-scores (30).

**Figure 5:**
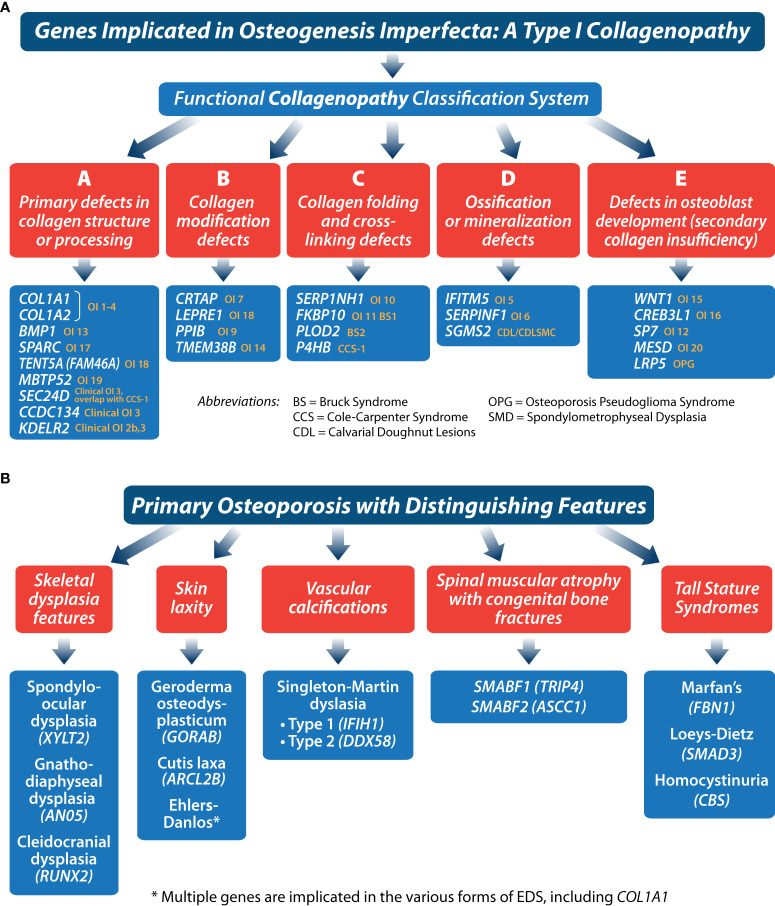
These figures **(A, B)** provide a framework for classifying primary osteoporosis presenting in childhood.

Advances in our understanding of the genetic basis of congenital bone fragility have been among the most important discoveries in the pediatric osteology field since the turn of the century, with numerous reviews describing what now approaches nearly two dozen monogenic causes of primary, juvenile-onset, monogenic osteoporosis (22, 31–34). In children with a significant fracture history but a negative work-up for an acute or chronic illness, the hunt for a genetic cause of osteoporosis is reasonable (even in the absence of hallmark signs such as blue sclerae or dentinogenesis imperfecta, which are not always present in primary osteoporosis). Children with heterozygous, loss of function mutations in *LRP5*, for example, present with clinically significant (long bone and vertebral) fractures, in the absence of classic OI features, and a small sub-group of patients with OI due to pathogenic variants in *COL1A1* and *COL1A2* will lack typical stigmata as well. A list of genes implicated in OI due to aberrant primary or secondary type 1 collagen function is shown in **Figure 5A**. Other (non-type I collagen) causes of primary osteoporosis in childhood are shown in **Figure 5B**.

Current genetic testing typically is based on sequence analysis of genomic DNA, to assess targeted gene panels or the whole exome. Although this approach is frequently successful, there are situations where the analysis of genomic DNA is not able to definitively establish a diagnosis; for example, variants affecting splice sites may be missed if they do not impact the splice consensus site, or they may be difficult to prove as having functional consequences. When analysis of genomic DNA is inconclusive, RNA sequencing may fill in the gaps. One of the challenges, though, is that genetic variants can only be detected and characterized by RNA sequencing if the genes of interest are expressed in sufficiently high quantities in the cells that are acquired from the patient. Recently, urine-derived stem cells from children and adolescents with suspected or confirmed OI were shown by the Rauch lab to produce type 1 collagen RNA in sufficient quantities to detect abnormal splicing in 7 of 8 patients with pathogenic or likely pathogenic variants in the splice site region or deep within the intron; abnormal deletions and duplications were also observed in urine-derived stem cell transcripts (35). Together, these results provided proof of principle that patients with negative type I collagen genetic testing on genomic DNA may nevertheless harbor type 1 collagen pathogenic variants with functional consequences.

**Figure 6:**
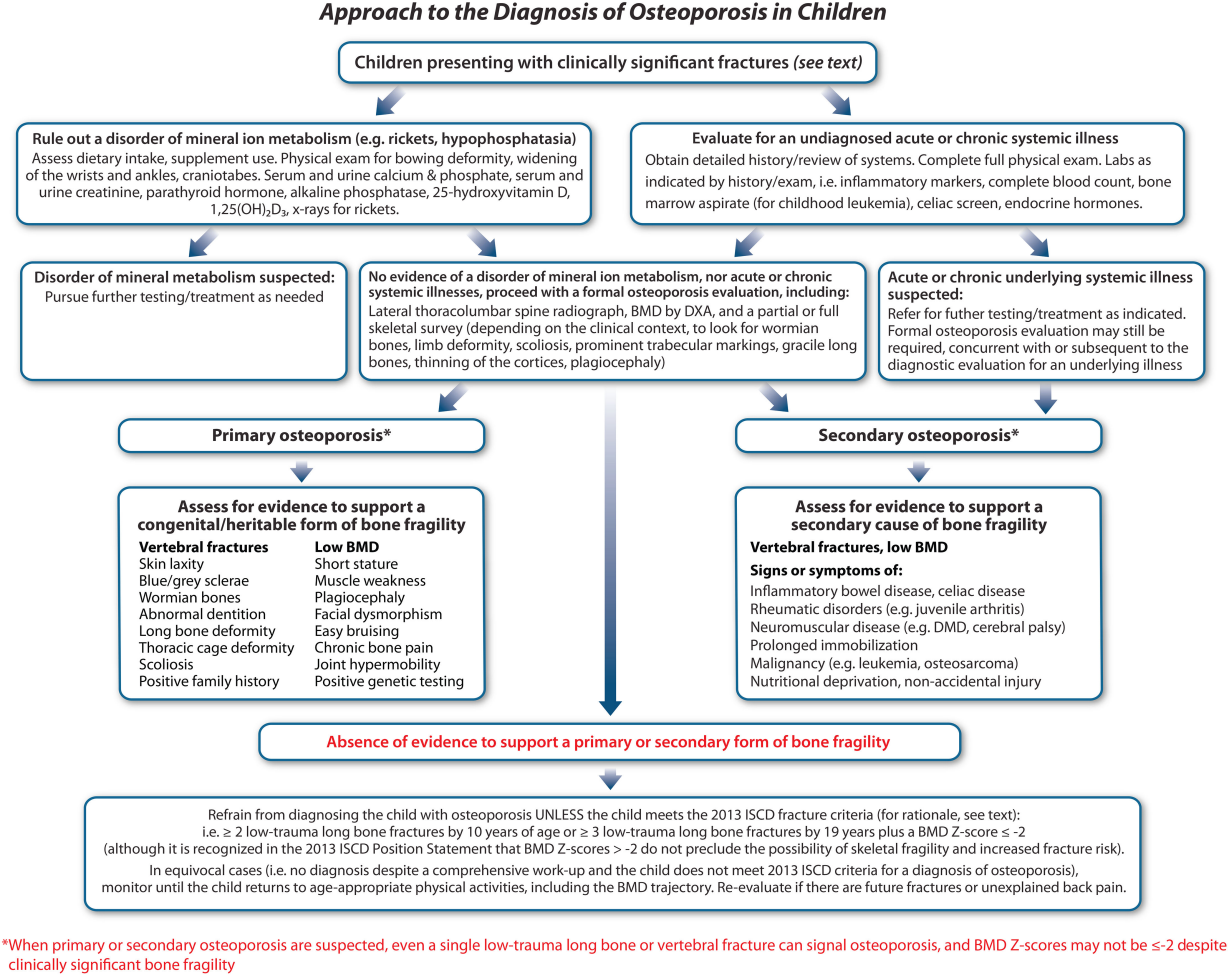
A proposed approach to the diagnosis of osteoporosis in children. After a careful diagnostic pathway, children will typically fall into one of three categories: a. primary osteoporosis due to a confirmed monogenic etiology, b. secondary osteoporosis due to an underlying medical condition or its treatment which predisposes to an increased risk of fractures, or c. “fractures in otherwise healthy children” (middle bottom). In cases of primary and secondary osteoporosis, even a single, low-trauma fracture may be sufficient to diagnose the child with osteoporosis, even if BMD Z-scores are normal. In the absence of a discrete etiology uncovered by the diagnostic process (middle bottom), a more conservative definition of osteoporosis is proposed in order to prevent children who were unfortunate during sports or play from being over-diagnosed with osteoporosis.

At the same time, it is recognized that not all clinicians have access to state-of-the-art genetic testing. In such cases, careful history and physical examination documentation combined with accurate interpretation of diagnostic imaging (radiography and DXA-based BMD) will form the basis for a clinical diagnosis of osteoporosis, as mapped out sequentially in this review. **Figure 6** provides an overview of the diagnostic pathway for children presenting with fractures at the pediatric osteology clinic.

### Step 3: Evaluate the child’s potential for “spontaneous” (i.e., medication-unassisted) recovery from bone fragility

**Figure 7:**
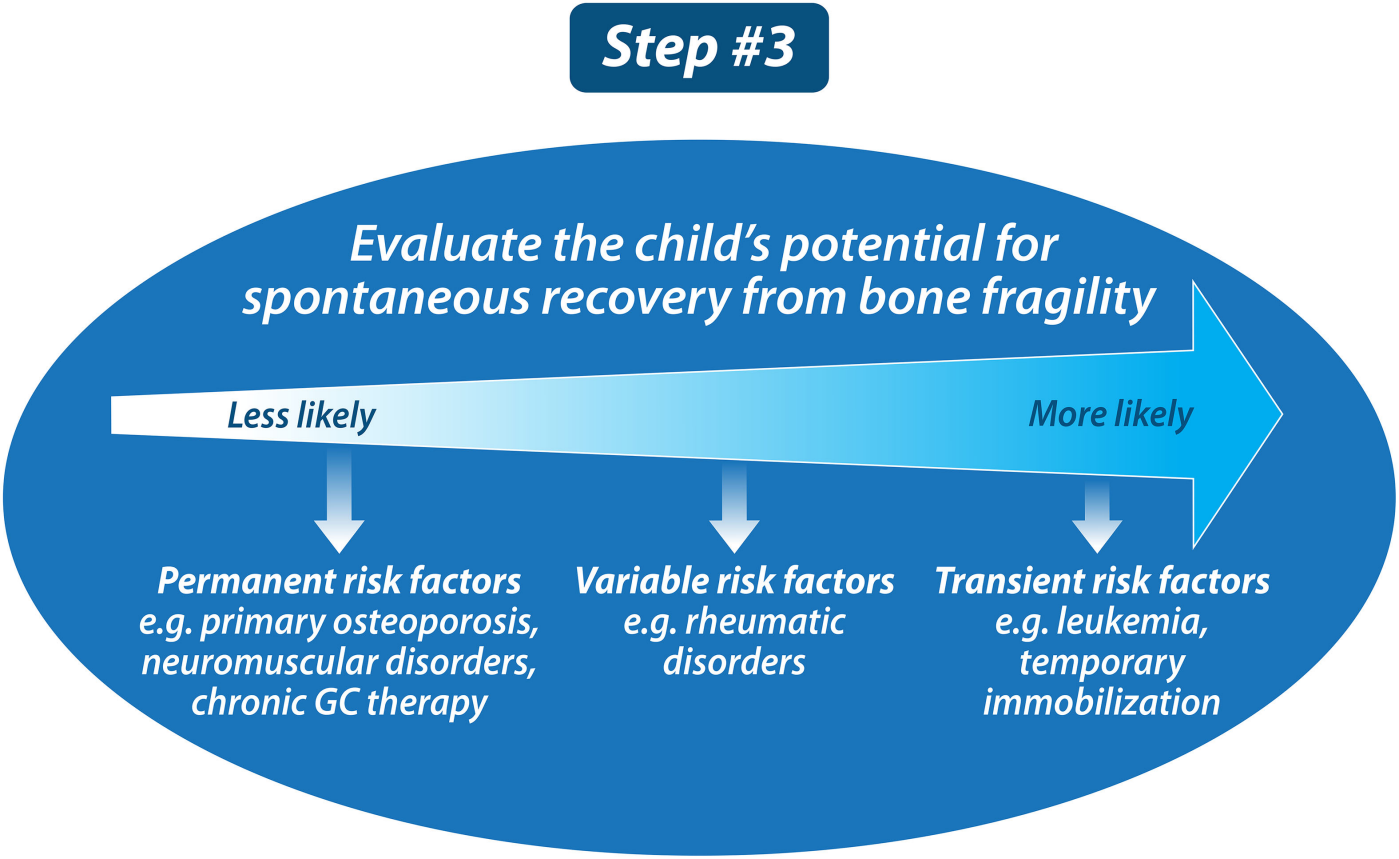
Once the child has been diagnosed with osteoporosis, the next step is to determine whether the child has the potential for “spontaneous, medication-unassisted recovery.” This is important because the pediatric skeleton has tremendous potential to recover from osteoporosis in those with waning risk factors plus residual growth potential.

Once the diagnosis of osteoporosis has been established in a given patient, the next step is to determine whether the child or adolescent has the potential to undergo spontaneous (i.e., medication-unassisted) recovery from bone fragility (Step 3, **Figure 7**). Unlike the adult (post-epiphyseal fusion) skeleton, the juvenile skeleton has tremendous potential to recover from osteoporosis, provided threats to bone health have abated and, relevant to vertebral fractures, there is sufficient residual growth potential. Indeed, recovery from osteoporosis does not only involve reclamation of BMD; restoration of normal vertebral dimensions (and thereby spinal height), a growth-mediated process, is also a key index of recovery.

The disease that best exemplifies structural recovery from bone health threats in the absence of bisphosphonate treatment is acute lymphoblastic leukemia. In this setting, vertebral body reshaping has even been observed while on leukemia therapy (13). The fact that vertebral body reshaping can take place while on leukemia therapy (which includes high-dose glucocorticoid (GC) treatment) is hypothesized to result from the intermittent nature of the GC prescription that is the backbone of current leukemia treatment protocols. By studying children with leukemia in the 6 years following diagnosis who had baseline or incident vertebral fractures, the Canadian STOPP Consortium showed using the Spinal Deformity Index (36, 37) that 77% of children had complete reshaping by their last follow-up visit, 18% had incomplete reshaping, and 5% had no change in their vertebral dimensions. Children with incomplete or absent vertebral body reshaping were older (on average 8 years of age at diagnosis, compared with 4.8 years in those with complete reshaping), and more frequently experienced moderate and severe collapse. In practical terms, these data revealed that younger children, and those with less severe collapse, appeared to reshape vertebral bodies more frequently in this context.

The long-term consequences of permanent vertebral deformity remain unknown. Adult studies have shown reduced quality of life due to chronic back pain, and also significant functional limitations (38, 39). Whether this is true in adults who experienced permanent vertebral deformity as children merits further study. In aging, vertebral fractures contribute to excess mortality (40) as well as restrictive pulmonary function compared to those without vertebral fractures (41), due to the well-known loss of spinal height in the elderly that is associated with vertebral collapse. Loss of vertebral height contributing to loss of linear height in children has also been suggested in pediatric leukemia (42), a clinical context where up to 1/3 of children will experience at least one vertebral fracture in the 6 years following diagnosis (13). Together, these adult studies suggest that permanent reductions in vertebral height sustained in childhood may have important consequences later in life. The GC-treated disease where this dialogue is particularly relevant is DMD, given the shortened lifespan due to cardiorespiratory failure. To date, there are no published reports of vertebral body reshaping without bisphosphonate therapy in pediatric DMD, an aggressive form of osteoporosis. This is likely because the GC prescription is long-term, growth is limited by the toxic effect of GC on the growth plate, and the myopathy is progressive.

**Figure 8:**
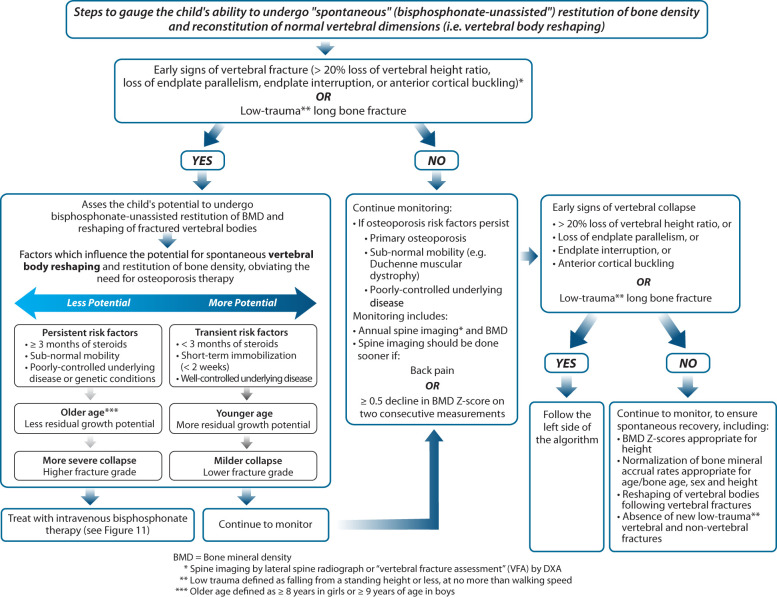
This figure provides an approach to gauge whether a child has the capacity to undergo spontaneous (medication-unassisted) recovery from osteoporosis, obviating the need for osteoporosis drug therapy.

Increases in bone turnover markers and improvements in BMD are also important signs of recovery. Thayu et al. (43) reported that reductions in bone turnover markers in pediatric Crohn’s disease were inversely associated with disease activity and that treatment with infliximab was associated with dramatic increases over one year. In childhood leukemia, studies have shown degrees of BMD restitution in the years after chemotherapy (44, 45). Cranial and spinal radiation predict a lack of BMD restitution, particularly at doses ≥ 24 Gy (45), related in part to growth hormone deficiency and short stature. In leukemia survivors, other reported risk factors for incomplete BMD restitution include vitamin D deficiency, hypogonadism, and reduced physical activity (46). In practical terms, pediatric osteologists look for normalization of the BMD Z-score for height as a sign of BMD restitution, along with a return to a normal rate of BMD accrual for age/pubertal stage and sex (47). Together, vertebral body reshaping, absence of new low trauma long bone fractures, and normalization of BMD for age, sex, and height are all indicators of recovery from osteoporosis in the pediatric setting. **Figure 8** outlines the criteria for gauging the child’s ability to undergo “spontaneous” BMD reclamation and reshaping of vertebral bodies following demonstrated bone fragility. Note is made of the fact that while the notion of vertebral body reshaping is largely restricted to the secondary osteoporosis setting (due to the potential for osteoporosis risk factors to abate), partial vertebral body reshaping has been described in a young patient with mild OI, providing proof of principle that milder forms of primary osteoporosis may also have some potential for recovery in the absence of bisphosphonate therapy (48).

### Step 4: Once a diagnosis of osteoporosis is established and it is estimated that the patient has limited potential for recovery, prepare the patient for (ideally intravenous) bisphosphonate therapy

**Figure 9:**
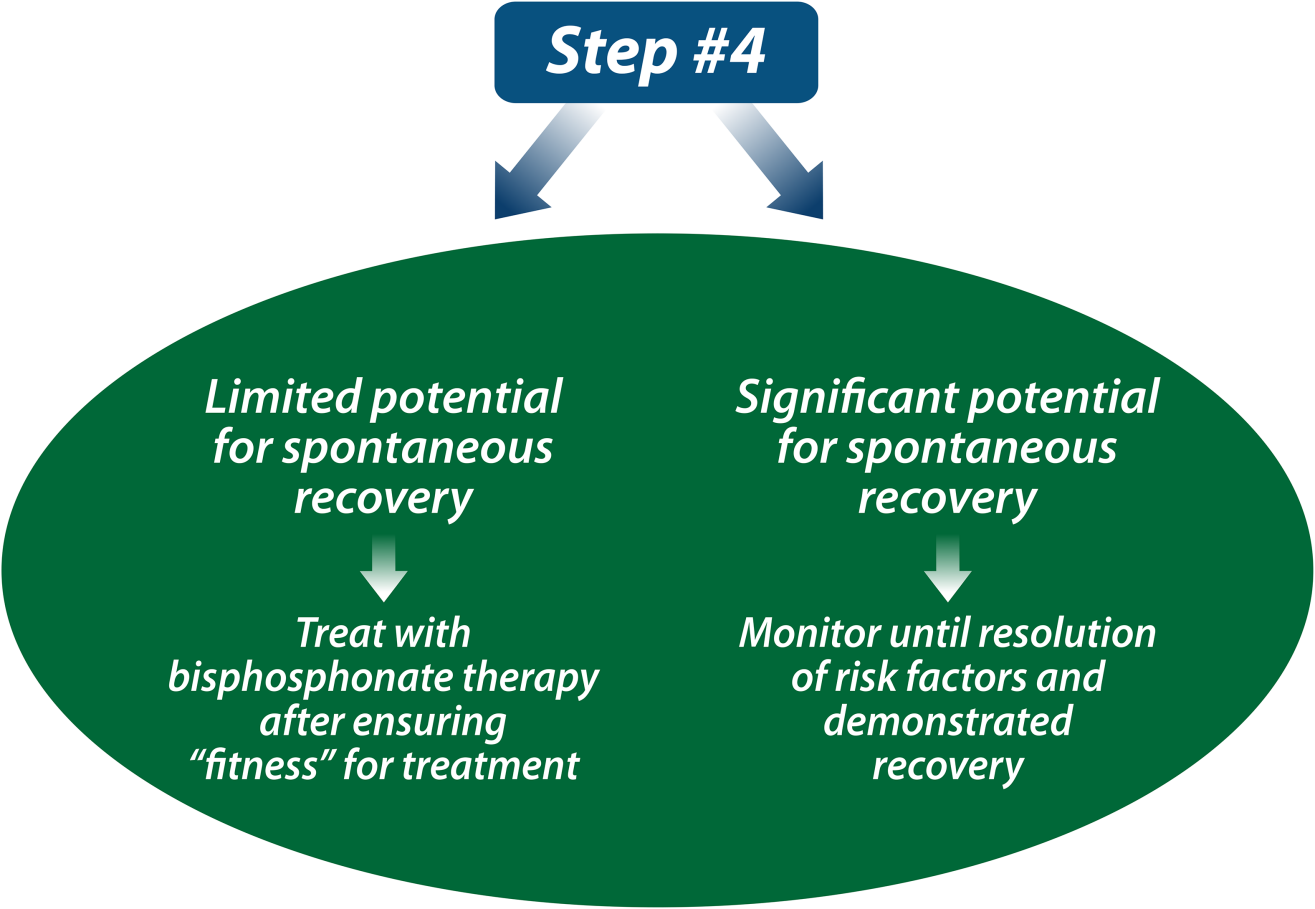
If a child has limited potential for spontaneous recovery from osteoporosis, the next step is to treat with the current standard of care, intravenous bisphosphonate therapy (or oral bisphosphonate therapy if intravenous is not available). If a child has significant potential for spontaneous reclamation of BMD and reshaping of vertebral bodies, the child can be monitored until resolution of risk factors and affirmation of recovery.

The decision to treat a child with a bisphosphonate determines the nature and frequency of follow-up. For children anticipated to recover from osteoporosis, annual follow-up for 1-2 years to affirm the anticipated, favorable prognosis is a reasonable approach (including BMD studies, lateral spine imaging if vertebral fractures were part of the osteoporotic phenotype, and a 25OHD level). For patients with limited potential for spontaneous recovery, treatment with intravenous bisphosphonate therapy is the standard of care (see Step 4, **Figure 9**). Importantly, a patient’s “fitness” for bisphosphonate therapy should be assessed prior to embarking on treatment, including adequate dietary and/or supplemental intake of calcium and vitamin D, euphosphatemia and eucalcemia, 25-hydroxyvitamin D sufficiency (serum 25OHD level at least 50 nmol/L or 20 ng/mL), and adequate renal function. IV zoledronic acid is contraindicated in patients with acute renal failure, and dose adjustments to IV therapy are required for patients with estimated glomerular filtration rates less than 60 ml/min/1.73 m^2^. Bisphosphonate-induced hypocalcemia is a well-known phenomenon; however, it is less appreciated that hypophosphatemia is also frequent post-bisphosphonate therapy, particularly in patients with chronic illness osteoporosis (49, 50), and may require phosphate supplementation to restore euphosphatemia. The first infusion side effects of IV bisphosphonate therapy can also precipitate adrenal insufficiency in patients on chronic GC therapy, even when they are taking supra-physiological doses. The reason for this is that although children with chronic illnesses may be receiving supra-physiological GC doses on a once-daily regimen for the treatment of their underlying illnesses, the GC duration of action may not be sufficient to cover the entire 24-hour period leading up to the next GC dose (51, 52). Therefore, it is prudent to recommend steroid stress dosing following the first dose of IV bisphosphonates in patients on chronic GC therapy, either prophylactically, or upon developing symptoms that would prompt such intervention in routine clinical practice (e.g., fever, vomiting, etc.) (53, 54).

The use of oral versus intravenous (IV) bisphosphonate therapy for pediatric osteoporosis has long been debated (55). Overall, IV pamidronate is the most extensively reported agent in children following the inaugural observational study in the late 1990s which showed improved pain, mobility, and reshaping of vertebral bodies on pamidronate in children with moderate to severe OI (56). Children were treated with cyclical, IV pamidronate at a dose of 9 mg/kg/year divided every 2 to 4 months for up to 5 years’ duration (56). IV zoledronic acid has since been introduced, given the advantage that it can be given over a shorter period of time and less frequently (57, 58); zoledronic acid is 100 times more potent than pamidronate (59). Both agents are nitrogen-containing bisphosphonates that inhibit farnesyl diphosphate synthase and thereby protein prenylation, a process crucial for osteoclast survival. A randomized study comparing pamidronate to zoledronic acid in OI showed that zoledronic acid had similar effects on LS BMD Z-scores and fracture rates over 12 months (57).

Of the oral agents, alendronate and risedronate have been the most extensively studied, with reports confirming that the oral bioavailability of alendronate in children is < 1%, similar to adults (60, 61). Given the low oral bioavailability of oral agents, it is not surprising that side effects are also reduced, though at an apparent cost to treatment efficacy (see next).

Despite the formidable challenges in conducting clinical trials among children with osteoporosis, there are now a number of randomized controlled trials of oral and IV bisphosphonates that have been carried out in primary (62–68) and secondary (5, 69–74) osteoporosis of childhood. Collectively, nearly all of the controlled IV *and* oral bisphosphonate therapy trials have shown significant increases in lumbar spine BMD Z-scores. The two routes of administration more clearly distinguish themselves, however, with respect to the direction of effect for fracture rates (recognizing that no studies were specifically powered to detect differences in fracture outcomes), changes in bone turnover (expected to decline on an effective anti-resorptive agent) and the capacity to bring about vertebral body reshaping (a key metric of a robust therapy in children, known to be particularly sensitive in children with OI) (75). Therefore, it is on *these* grounds that we adjudicate the response to IV versus oral bisphosphonate therapy.

Based on observational studies, it is expected that fractured vertebral bodies will undergo reshaping with bisphosphonate therapy (58, 75–77), thereby providing a key index of benefit (provided the child is growing). The controlled trials to date in growing children with OI that quantified vertebral body height clearly showed increases in those receiving IV bisphosphonate therapy (67, 78, 79), whereas none of the controlled oral bisphosphonate studies in OI where it was measured showed a positive effect on vertebral height (62–64, 80). In fact, vertebral fracture rates were more frequent in patients with OI on risedronate (62), and bone turnover markers increased (instead of the expected decrease) on risedronate in a study of children with rheumatic disorders by Rooney et al. (70). In a large, randomized trial of daily oral alendronate for moderate and severe pediatric OI (66), there was no effect of alendronate on the cortical width of trans-iliac specimens. In contrast, this is a key structural index derived from a precise measurement with a known positive response to IV bisphosphonate therapy in OI (81). Another compelling observation that supports IV over oral therapy is from a controlled OI trial (64), where risedronate did not lead to an increase in the trabecular volumetric BMD at the distal radius compared to placebo; on the other hand, IV therapy caused significant increases in BMD at this site (82). Overall, these data support the use of IV instead of oral bisphosphonate therapy first-line; the increases in BMD on oral agents nevertheless suggest that oral may be a reasonable therapy in situations where IV bisphosphonates are unavailable.

### Step 5: Start (ideally intravenous) bisphosphonate therapy at initiation doses, followed by dose titration to achieve normal BMD Z-score trajectories for age, sex, and height. Aim to discontinue therapy at the time of epiphyseal fusion in children with primary osteoporosis or following the resolution of risk factors in children with secondary osteoporosis

**Figure 10:**
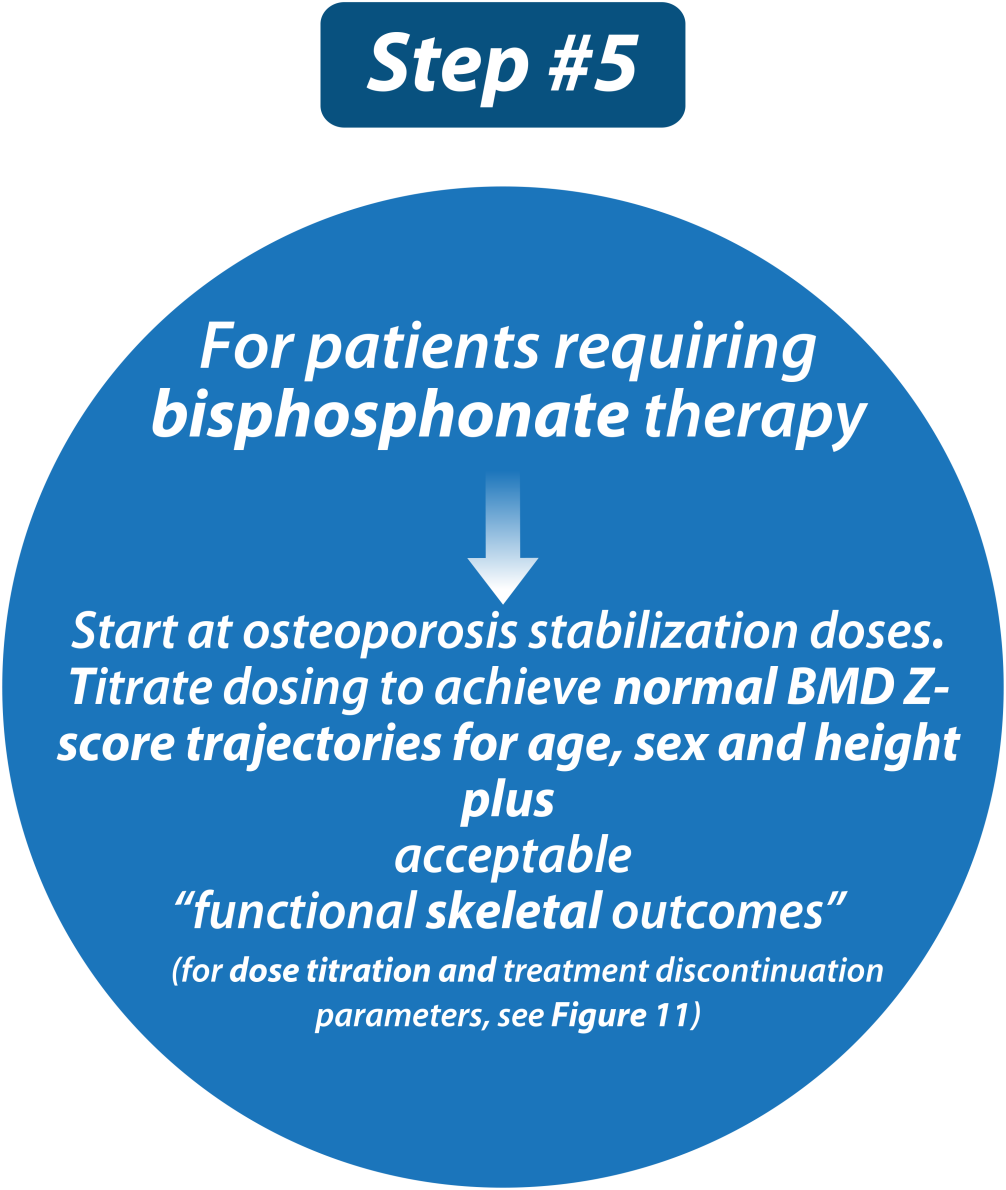
Children with osteoporosis should be treated with bisphosphonate therapy at published doses and in specialized centers by practitioners with experience in the care of such children.

**Figure 11:**
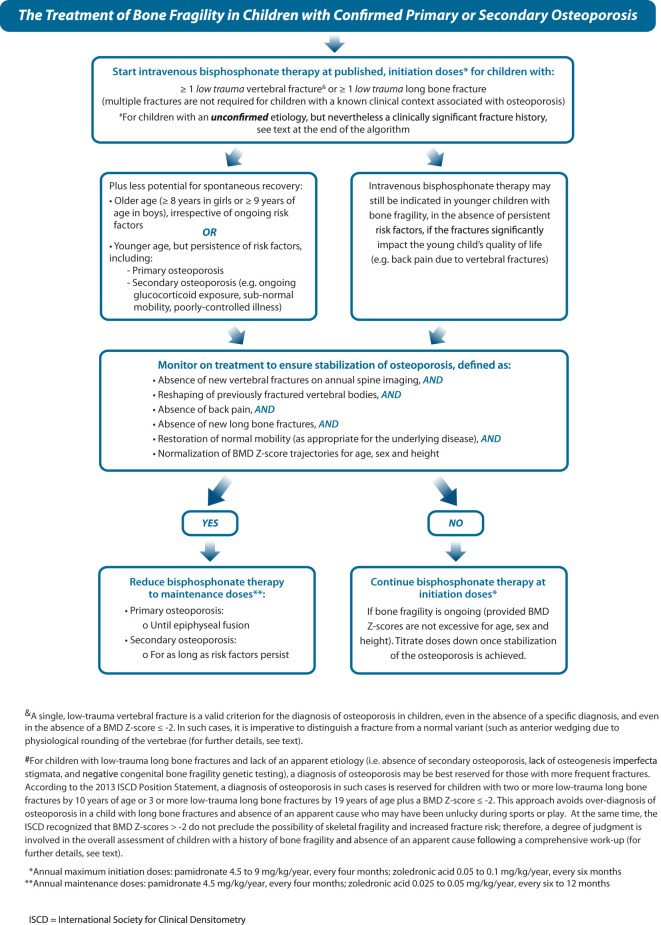
This figure provides an algorithm for the treatment of osteoporosis with intravenous bisphosphonate therapy, the standard of care, including initiation doses (during the stabilization phase) plus dose titration (during the maintenance phase).

**Figure 10** (Step 5) and **Figure 11** provides a practical algorithm for the treatment of primary and secondary osteoporosis in childhood. A frequently prescribed IV bisphosphonate regimen is cyclical IV pamidronate (maximum dose 9 mg/kg/year for children ≥ 3 years, 3 mg/kg divided equally over 3 days given every 4 months) (56, 83–86). Due to high bone turnover in younger children, pamidronate is dosed more frequently (2.25 mg/kg divided equally over 3 days, every 3 months for children 2 to 3 years of age, and 1.5 mg/kg divided equally over 3 days, every 2 months to children < 2 years of age). Zoledronic acid is increasingly used in clinical care due to the ease of less frequent dosing intervals and shorter infusion time compared to pamidronate (maximum dose 0.1 mg/kg/year given as two equal doses (0.05 mg/kg) every 6 months in children ≥ 2 years, and 0.025 mg/kg every 3 months in children < 2 years) (57, 87, 88). Some clinicians favor a lower annual starting dose (such as a single-day pamidronate infusion of 1 mg/kg every 3 months, 4 mg/kg/year) (89, 90). Apart from these regimens, other IV doses and intervals have also been reported, though none has gone head to head in controlled, comparative trials, the exception being pamidronate versus zoledronic acid which showed similar effects on BMD and fracture rates in OI (57). With such little controlled comparative data, it is impossible to state which IV agents and intervals achieve the best results for mitigating fractures and pain and improving overall function. Regardless, bisphosphonate therapy should only be administered by clinicians with the appropriate expertise and infrastructure to support peri-infusion care, and the maximum, published annual doses should not be exceeded so as to avoid iatrogenic osteopetrosis arising from toxic doses (91).

The approach to dose adjustments and the duration of bisphosphonate therapy are also questions frequently posed by pediatricians. A number of key observations unique to children have influenced practice in this regard. The first observation has led to continuing bisphosphonate therapy until final height attainment in those with permanent or persistent risk factors, as follows. Among children with open epiphysis and therefore ongoing endochondral bone formation, following treatment discontinuation the newly formed bone adjacent to the growth plate will be “treatment naïve” and thereby lower density, creating a stress riser between high (previously treated) and low (untreated) density bone (82). Not surprising, metaphyseal fractures have occurred post-bisphosphonate discontinuation in children with OI who are still growing, since the newly formed bone will invariably be low density (92). In fact, metaphyseal fractures have even occurred *during* intermittent IV bisphosphonate therapy at the interface between the dense metaphyseal lines created at the time of therapy and the (2 mm) adjacent treatment-naïve bone (93). This latter report raises the question of whether IV bisphosphonates should be administered with as short an infusion interval as possible, a line of thinking that is challenged by the demands on the patient and the health care system arising from frequent infusions.

Further support for continuation of therapy to final height in those with persistent or permanent risk factors arises from a study by Rauch et al. (92). These investigators showed using pQCT that there were significant declines in trabecular BMC Z-scores at the distal radius following pamidronate discontinuation in children with OI who were still growing. On the other hand, discontinuation after epiphyseal fusion was associated with more stable BMD Z-scores 2 years later. Balancing these observations with lingering concern about over-suppression with longer-term therapy, the current recommended approach is to treat patients initially with a higher dose regimen until the patient is clinically stable. Typically, this equates to a minimum of 2 years, the time point at which the maximum benefit from bisphosphonate therapy has been observed in children with OI (81). Once the patient is clinically stable, a lower (half-dose or less) (75, 94) maintenance protocol is given until the patient attains final adult height, at which time treatment can be discontinued if the patient is stable (75). The goal of the maintenance phase of therapy in children with permanent or persistent risk factors is to preserve the gains realized during high-dose therapy while avoiding over-treatment (75, 94). To this end, the dose of IV bisphosphonate therapy in the maintenance phase may require further downward titration to avoid unnecessarily high BMD Z-scores – this can be achieved by decreasing the dose or by increasing the interval between infusions. Palomo et al. (75) recently reported that long-term (at least 6 years) bisphosphonate therapy with downward dose titration in pediatric OI led to higher BMD Z-scores compared to historical controls, and also resulted in vertebral body reshaping, although it was notable that non-VF rates were still high (reduced by 50% by on a high pre-treatment fracture burden) and most patients developed scoliosis. An outstanding question about the duration of therapy in those who stop around the time of adult height attainment but have persistent risk factors for fractures (e.g. OI, ongoing GC exposure) is whether they will require re-introduction of bisphosphonate therapy in the adult years and, if so, at what time point.

In children with resolution of risk factors during growth (i.e., cessation of GC therapy, remission of inflammation, recuperation of mobility), discontinuation of therapy can be considered once the child has been fracture-free (VF and non-VF) for at least 6 to 12 months, previously fractured vertebral bodies have stabilized or undergone reshaping and BMD Z-score velocities are appropriate for age, sex, and height. Re-introduction of therapy may be required during growth if the prior risk factors for osteoporosis recur and patients once again meet the criteria for treatment initiation.

The long-term effects of bisphosphonate therapy have been discussed in detail previously (95). In brief, despite decades of searching for osteonecrosis of the jaw in children on bisphosphonate therapy, to date, there are no reports of this phenomenon in the childhood and adolescence settings (50, 96). Nevertheless, a proactive approach to dental surveillance and home oral care is advised (97). Similarly, a review of femur fractures with atypical characteristics in children with OI by two different groups showed that such fractures were no more frequent on bisphosphonate therapy; instead, both groups concluded that femur fractures with atypical features were related to the severity of the OI (98, 99). While the timing of dental development is not delayed in children with OI who have received bisphosphonate therapy, IV bisphosphonates have been associated with delayed osteotomy healing (100). To mitigate this phenomenon, Anam showed that a combination of delaying bisphosphonate infusions until there is sufficient callus formation (usually 4 months following surgery) plus using an osteotome instead of a power saw are effective in reducing the frequency of delayed osteotomy healing (101).

## Unmet needs and future directions

### The need for an efficacious osteoporosis agent with a convenient route of administration and favorable side effect profile

While there is now higher quality evidence for the use of intravenous bisphosphonate therapy in both primary and secondary osteoporosis of childhood (as discussed earlier), the first-infusion side effects of IV bisphosphonate therapy, along with the inconvenience of the IV route, have spurred interest in alternative forms of anti-resorptive therapy. RANKL is a critical mediator of osteoclast formation, function, and survival (102), and denosumab is a fully human, monoclonal antibody that targets RANKL to prevent the activation of RANK, thus inhibiting cortical and trabecular bone resorption without directly interacting with the skeletal surface (103). Large studies in adults showed that denosumab 60 mg every 6 months reduces hip, vertebral, and non-vertebral fracture rates compared with placebo, in the absence of an increased frequency of adverse events (104). Other adult studies have also confirmed that adverse events with denosumab are similar to an active comparator (oral alendronate), including the frequency and magnitude of hypocalcemia (104, 105). Denosumab is approved for osteoporotic men and post-menopausal women with a high risk for fracture; it is also approved for adult GC-induced osteoporosis.

In children, however, the denosumab-related “rebound” or “overshoot” phenomenon, previously mainly characterized in adults as loss of BMD and an increase in vertebral fractures following denosumab discontinuation (106), appears amplified due to its association with severe (even life-threatening) hypercalcemia (107). In a variety of disease states in which denosumab has been used in children including classic forms of OI, OI type VI, giant cell tumors, aneurysmal bone cysts, and fibrous dysplasia (108–113), the hypercalcemic rebound phenomenon has occurred both following denosumab discontinuation and while on active treatment. In children with surgically-unresectable giant cell tumors, aneurysmal bone cysts, and fibrous dysplasia lesions, the benefits of denosumab may outweigh the risks, although studies are needed to determine the optimal dosing regimens to achieve maximum benefit and to minimize to side effects (including the rebound phenomenon). While a moratorium has essentially been placed on denosumab in classic forms of OI, there does appear to be rationale for its use in OI type VI in combination with IV zoledronic acid as alternating therapy (the latter, to prevent the rebound phenomenon, while allowing the child to benefit from the denosumab otherwise) (110). OI type VI is an unusual form of the condition whereby the abnormal (osteomalacic) bone is hypothesized to interfere with bisphosphonate adherence to the bone surface, which in turn may result in reduced bisphosphonate efficacy (114). A recent report describing alternating short- (denosumab) and long- (zoledronic acid) acting anti-resorptive therapy in a child with OI VI was successful in mitigating the rebound phenomenon, thereby allowing the child to benefit from the potent anti-resorptive effect of denosumab (110).

Since the rebound phenomenon arises from exuberant skeletal resorption following reactivation of osteoclasts when the effect of the antibody wanes, it is unclear whether this phenomenon will be a concern in low bone turnover states such as pediatric GC-induced osteoporosis. On the other hand, rebound may theoretically occur when bone turnover increases at the time of spontaneous or induced puberty, or following GC cessation. As such, denosumab in low bone turnover states merits careful study in clinical trials and should not be administered outside of highly specialized pediatric osteology clinics. Since RANKL is also implicated in the inflammatory pathway that contributes to muscle destruction in DMD (115), studies assessing the impact of denosumab on *muscle* strength in DMD show promise in pre-clinical models (116).

### Prevention of first-ever fractures using drugs that are anabolic to bone and sequential therapy

The BMD of trabecular-rich bone such as the spine is more readily modifiable by anti-resorptive therapy than cortical bone, because porous (spongy) bone has greater surface area to accommodate a bone density-altering therapy compared with compact bone. In addition, while anti-resorptive therapy increases the cortical width of long bones in children who are growing, the reductions in long bone periosteal circumference that are germane to diseases like DMD and OI are not modifiable by any therapy that acts only on endocortical and trabecular surfaces (such as anti-resorptive drugs); therefore, medications are needed that also target periosteal apposition.

As such, the door is decidedly open for novel therapies that are anabolic to bone, and which would be ideal in children with a need for prolonged GC therapy, with poor growth, and with reductions in periosteal circumference (the latter whether due to myopathies as in DMD or due to the periosteal apposition-limiting effects of prolonged GC). The overall goal of such an approach would be to prevent first-ever fractures in children with the greatest risk of bone fragility and the least potential for recovery.

Teriparatide, recombinant human PTH (1–34), is approved by the United States Food and Drug Administration (FDA) for initial treatment of post-menopausal osteoporosis with a high risk of fracture for patients who have failed prior osteoporosis therapy and for adults with GC-associated osteoporosis (117). Teriparatide reduces the risk of VF and non-VF in post-menopausal women; the effect on fractures of the hip was inconclusive due to a low incidence of hip fractures in a large, randomized controlled trial (118). Overall, teriparatide positively affects spine BMD, but not BMD at the hip or forearm (118). Where children are concerned, this anabolic drug recently had an FDA black box warning lifted, initially in place due to the development of osteosarcoma in growing rats treated at doses that were three to 50 times higher than human, adult equivalents (119). Subsequent studies in the same strain of rats found no evidence of malignancy with doses that were three times higher than the human equivalent (120).

BMD declines rapidly in the 12 months following teriparatide cessation, although fracture reductions persist for up to 2 years (121). Teriparatide, followed by alendronate, mitigates this loss (122). A recent case report of teriparatide in a 20-year-old man with DMD described improvement in back pain due to VF after 6 months of teriparatide, plus increases in LS BMD, improvement in quality of life, and increases in suppressed bone biomarkers (123). These findings support further study of PTH in DMD post-epiphyseal fusion. On the other hand, the impact of PTH on bone is attenuated in adults when administered after bisphosphonate therapy (124). This may undermine its use in men with DMD who received bisphosphonates in childhood.

Sclerostin is a potent inhibitor of bone formation, secreted by the osteocyte to inhibit the anabolic Wnt signaling pathway. Sclerostin antibody therapy releases the inhibition on sclerostin-mediated bone formation and has been approved for women with post-menopausal osteoporosis (romosozumab). Mice treated with anti-sclerostin antibody show not only increases in BMD and bone formation markers but also positive changes in bone geometry (including increases in periosteal circumference) (125). In adults receiving anti-sclerostin antibody, bone formation returns to baseline by about six months after the first sub-cutaneous injection, and subsequent doses appear to have less of a beneficial effect on bone formation (126). As a result, it has been recommended to “seal in” the gains of sclerostin antibody treatment with a sequential therapy approach using an anti-resorptive treatment (126, 127). Anti-sclerostin antibody (BPS804) has been used in a phase 2 study of adults with OI (128), showing a significant increase in markers of bone formation and reduction in bone resorption. International pediatric OI trials are now underway investigating the use of BPS804 in this setting (clinicaltrials.gov NCT05768854, and NCT05125809).

### Prevention of first-ever fractures in high-risk settings and treatment of residual bone health deficits following major threats to bone health

The time is nigh to focus efforts on prevention of first fractures in high-risk settings. Children with OI who are in the greatest need of prevention of first fractures typically present with bone fragility in the first few years of life; as a result, treatment at a young age/early stage is already often inherent to this genetic bone fragility condition. On the other hand, fracture rates increase precipitously in boys with DMD following GC initiation (which typically starts around 4 to 6 years of age), with an ongoing high probability of fractures thereafter (129). Death due to fat embolism syndrome has been described following fractures in this setting (130), and long bone fractures are associated with premature, permanent loss of ambulation (10). Recent observations have provided insight into the clinical variables that are associated with bone loss and vertebral fractures in DMD, including systemic signs of GC exposure such as bone age delay, short stature, and excess weight (131) as well as loss of ambulation (132). These characteristics will guide future efforts to identify the best candidates for early anticipatory osteoporosis prevention in DMD and other conditions with a high probability of childhood fractures.

Another clinical scenario that prompts consideration for a modified approach is patients who have escaped significant enough bone fragility during chronic illness management to dodge osteoporosis therapy, but who nevertheless are left with residual BMD deficits: examples include leukemia and other cancer survivors, and adolescents with chronic GC administration. In order to optimize BMD accrual during the critical years of bone mass acquisition, the question has been raised whether IV (or oral, given the lack of overt bone fragility) bisphosphonate therapy may reduce the reported increased fracture rates later in life (133, 134).

With these unmet needs in mind, the pediatric osteology field has its work cut out for the next decade on a background of significant progress already made in osteoporosis diagnosis, monitoring and treatment since the most recent turn of the century.

## Author contributions

LW: Conceptualization, Data curation, Formal analysis, Funding acquisition, Investigation, Methodology, Project administration, Resources, Software, Validation, Visualization, Writing – original draft, Writing – review & editing.
